# The Impact of Co-occurring Post-traumatic Stress Disorder and Substance Use Disorders on Craving: A Systematic Review of the Literature

**DOI:** 10.3389/fpsyt.2021.786664

**Published:** 2021-12-14

**Authors:** Fabien Renaud, Louise Jakubiec, Joel Swendsen, Melina Fatseas

**Affiliations:** ^1^Pôle Inter-établissement d'addictologie, CHU de Bordeaux et Centre Hospitalier Charles Perrens, Bordeaux, France; ^2^University of Bordeaux/CNRS-UMR 5287, Bordeaux, France; ^3^EPHE, PSL Research University, Paris, France

**Keywords:** post-traumatic stress disorder (PSTD), substance use disorder, dual disorder (DD), craving, integrated treatment, systematic (literature) review

## Abstract

The frequent co-occurrence of post-traumatic stress disorder (PTSD) and substance use disorders (SUDs) leads to manifestations of both conditions that are more severe and more resistance to treatment than single disorders. One hypothesis to explain this synergy is the impact of intrusive memories on craving which, in turn, increases the risk of relapse among patients with substance use disorders. The aim of this systematic review is to examine this possibility by assessing the impact of PTSD and its symptoms on craving among dual disorder patients. Using PRISMA criteria, four databases were comprehensively searched up to June, 2021, in order to identify all candidate studies based on broad key words. Resulting studies were then selected if they examined the impact of PTSD or PTSD symptoms on craving, and if they used standardized assessments of PTSD, SUD, and craving. Twenty-seven articles matched the selection criteria and were included in this review. PTSD was found to be significantly associated with increased craving levels among patients with alcohol, cannabis, cocaine, tobacco, and other substance use disorders. Exposition to traumatic cues among dual disorder patients was also shown to trigger craving, with an additive effect on craving intensity when exposure to substance-related cues occurred. In addition, certain studies observed a correlation between PTSD symptom severity and craving intensity. Concerning mechanisms underlying these associations, some findings suggest that negative emotional states or emotion dysregulation may play a role in eliciting craving after traumatic exposure. Moreover, these studies suggest that PTSD symptoms may, independently of emotions, act as powerful cues that trigger craving. These findings argue for the need of dual disorder treatment programs that integrate PTSD-focused approaches and emotion regulation strategies, in addition to more traditional interventions for craving management.

## Introduction

The diagnosis of Post-Traumatic Stress Disorder (PTSD) first appeared in the 3rd edition of the Diagnostic and Statistical Manual of Mental Disorders in 1980 (1), based largely on clinical descriptions of soldiers returning from the Vietnam war (2). Its definition has evolved considerably over recent decades, including its removal from the anxiety disorders in DSM 5 as well as the creation of a distinct diagnostic category for this disorder (3). PTSD is characterized by a variety of symptoms that persist over the months or years following a traumatic event and that notably include intrusive memories, avoidance of cues associated with the event, alterations of cognition and mood, and a state of hyperarousal. Although diverse mental disorders are frequently associated with PTSD, substance use disorders (SUDs) are particularly prevalent (4–6). The principal hypotheses that have been formulated to explain these associations include self-medication (implying that PTSD is the primary condition and that substance use disorders occur later), the notion that addiction to substances may constitute a risk factor for the occurrence of traumatic events (whereby PTSD is a secondary condition), and finally the possibility that both disorders share common vulnerability factors (7). Regardless of which mechanism best explains these forms of dual disorder, the combination of PTSD and addiction leads to poorer prognosis, increases in suicide attempts, greater social disability, poorer treatment adherence, and reduced medication efficacy when compared to patients without comorbidity (8–10). In line with these results, a recent review of clinical investigations also documented a strong relationship between the diagnosis of PTSD and increased substance use and relapse in dual disorder individuals (11), but again the exact mechanisms underlying this association remain unclear.

Craving has been studied extensively over the years and particularly over the past two decades due to acknowledgment of its crucial role in addiction (12, 13). Craving refers to the intense, urgent, and unwanted desire to consume a substance (14) and it is now considered to be a core component of addiction with important diagnostic implications following its inclusion in DSM-5. Based on findings that demonstrate a prospective link between craving episodes and substance use, craving is increasingly viewed as a central construct in the etiology and course of different forms of addiction, and it is a strong predictor of treatment outcome (15–19). Among the diverse factors that may affect craving, a large body of research has highlighted the major role of substance-related cues and stress (20–29). These investigations have shown the ability of substance-related cues and stress exposure to elicit craving among individuals with alcohol, opiate, cocaine, tobacco, and cannabis dependence. Moreover, laboratory studies have also shown that exposure to stress-related events among individuals with alcohol use disorder (AUD) reliably elicits craving in a manner that is as powerful as alcohol-related cues (30, 31). Although similar patterns of reactivity have been shown among individuals with PTSD and alcohol use disorder after exposures to personalized trauma cues *via* “trauma scripts,” such scripts were found to provoke greater craving that non-trauma scripts and to be more salient in eliciting alcohol craving (31, 32). These results could suggest that the intrusive memories experienced by persons with PTSD and the significant stress they induce may therefore constitute major triggers of craving as well as explain reductions in treatment efficacy in this population. This pattern of findings is consistent with the findings that patients in SUD treatment who report higher PTSD scores also report higher scores on craving, depression, anxiety and stress (33), with a potential relationship between PTSD severity, SUD severity and craving levels.

One hypothesis to explain the synergy of PTSD and SUDs as a dual disorder is therefore the impact of intrusive memories or trauma-related cues on craving which, in turn, increases the risk of relapse among patients with substance use disorders. Examining this relationship across different forms of substance addiction should help elucidate the mechanisms underlying the general increase in clinical severity in this population, and the literature on this topic is now of sufficient size to permit a reliable summary that should more fully respond to the goals of precision psychiatry and personalized medicine (34). The aim of this systematic review is to address this issue by assessing all published investigations of the impact of PTSD and its symptoms on craving, among dual disorder patients.

## Methods

### Research Design

The study involved a systematic review of the literature based on the Preferred Reporting Items for Systematic reviews and Meta-Analyses (PRISMA) guidelines (35).

### Information Sources

This review was based on the following databases: PUBMED/MEDLINE, Psychinfo, Cochrane, and Wiley Online Library. The search was performed for all years up to June, 2021.

### Search

The following search terms were used:

[(≪ Addiction ≫ or ≪ Substance Use Disorder ≫ or ≪ Substance-related Disorder ≫) and (≪ Post Traumatic Stress Disorder ≫ or ≪ PTSD ≫) and (≪ Craving ≫ or ≪ Urge ≫)].

### Eligibility Criteria

The following criteria were used to select investigations for this review:

1. Studies Published in English-Language peer-reviewed journals.
2. Studies concerning patients, with no restrictive criteria regarding age, sex, ethnic origin, or place of residence. Studies had to include participants with PTSD and SUD comorbidity, defined, or explored according to standardized questionnaires.
3. Studies including measures of craving, and assessing the impact of PTSD or PTSD symptoms on craving occurrence or severity. It was not necessary that craving was designated as the primary outcome of the study in order for it to be included in this review.

Studies were excluded if they were based on animal models, or if they were limited to conference abstracts, dissertations, book chapters, or incomplete articles.

### Study Selection

Two authors independently examined all titles and abstracts. Relevant articles were obtained in full-text and assessed for inclusion criteria separately by the two reviewers based on the inclusion and exclusion criteria previously mentioned. Disagreements were resolved *via* discussion of each article for which conformity to inclusion and exclusion criteria were uncertain and a consensus was reached. The reference lists of major papers were also manually screened in order to ensure comprehensiveness of the review. All selected studies were read in full to confirm inclusion criteria, study type, and study population.

### Quality Assessment

Two reviewers (LJ and MF) assessed the quality of data in the included studies using the Quality Assessment Tool for Observational Cohort and Cross-Sectional Studies (S2C) from National Institutes of Health (36). This tool is comprised of 14 questions with responses to each being “yes,” “no,” or “other” (not applicable, NA or nor reported, NR). We rated the overall quality of each included study as “good,” “fair,” or “poor.”

### Collecting Data

Sample characteristics (including socio-demographic data, comorbidity, and treatment status), and information on study design and methods of assessment of PTSD, SUD, and craving were extracted. Table 1 presents these data extracted from the selected studies.

**Table 1:** Data items extracted from the selected studies.

**Evaluation criteria**	**Variables collected**
**Study characteristics**	Retrospective, prospective or cross-sectional observational studies. (Or) experimental studies: comparative or not, exposure to stimuli (substance-related cues, trauma, and stress) (Or) systematic review or meta-analysis
**Sample characteristics**	Socio-demographic characteristics (age, sex, employment status, income, and education level), treatment (inpatient, outpatient, no treatment), type of SUD, and comorbidity
**Evaluation methods**	DSM diagnostic criteria and/or evaluation scales for the different variables of interest: diagnosis and level of severity of SUD and PTSD, craving (frequency and intensity)
**Results**	Presented according to substance type

## Results

### Study Selection

**Figure 1:**
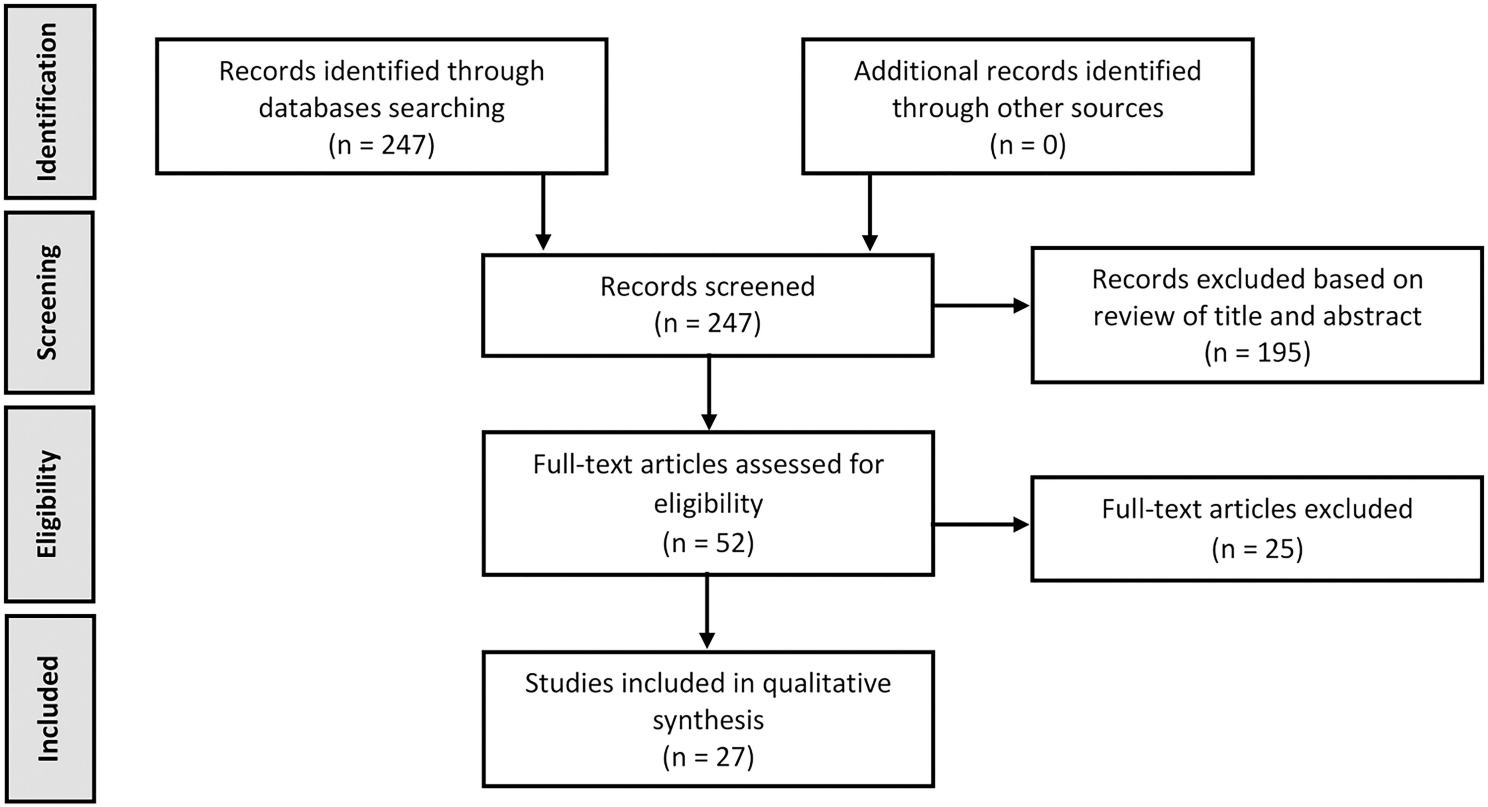
PRISMA flowchart of selected abstracts and articles.

A total of 247 articles were identified through the search of the databases. After review of titles and abstracts, 52 articles were selected for further examination. After reading the full text, 27 met inclusion criteria for this review. This process is described in the PRISMA flowchart (Figure 1). The selected articles were published between 2002 and 2021.

### Quality Assessment

A summary of risk of bias is presented in Table 2. Eleven studies were considered to be of “good” quality, six were “fair” quality and nine of “poor” quality.

**Table 2:** Overall quality rating of the included studies using the The National Institutes of Health quality assessment tool for observational cohort and cross-sectional studies.

**Study**	**Q1**	**Q2**	**Q3**	**Q4**	**Q5**	**Q6**	**Q7**	**Q8**	**Q9**	**Q10**	**Q11**	**Q12**	**Q13**	**Q14**	**Quality**
Coffey et al. (32)	Y	Y	NR	Y	N	Y	Y	Y	Y	N	Y	N	NA	Y	Good
Saladin et al. (47)	Y	Y	NR	Y	N	Y	Y	Y	Y	N	Y	N	NA	Y	Good
Brady et al. (41)	Y	Y	NR	Y	N	Y	Y	Y	Y	Y	Y	N	Y	Y	Good
Schumacher et al. (42)	Y	Y	NR	N	N	N	Y	N	Y	N	Y	N	N	N	Poor
Coffey et al. (30)	Y	Y	NR	Y	N	Y	Y	Y	Y	Y	Y	N	N	N	Good
Beckham et al. (43)	Y	Y	NR	Y	N	Y	Y	Y	Y	Y	Y	N	N	Y	Good
Driessen et al. (58)	Y	Y	Y	Y	N	NA	N	NA	Y	NA	Y	NA	NA	Y	Fair
Coffey et al. (31)	Y	Y	NR	Y	N	Y	Y	Y	Y	Y	Y	N	NA	Y	Good
Drapkin et al. (51)	Y	Y	NR	Y	N	NA	NA	NA	Y	N	Y	NA	NA	N	Poor
Jayawickreme et al. (50)	Y	Y	NR	Y	N	NA	NA	NA	Y	NA	Y	N	NA	N	Poor
Nosen et al. (38)	Y	Y	NR	Y	N	Y	Y	Y	Y	Y	Y	N	NA	N	Good
Simpson et al. (52)	Y	Y	NR	N	N	Y	Y	NA	Y	Y	Y	NA	Y	N	Fair
Dedert et al. (44)	Y	Y	NR	Y	N	Y	Y	Y	Y	N	Y	N	NA	N	Fair
Boden et al. (56)	Y	Y	NR	Y	N	NA	NA	Y	Y	NA	Y	NA	NA	Y	Fair
Tull et al. (45)	Y	Y	NR	N	N	Y	Y	Y	Y	N	Y	N	NA	Y	Fair
Dedert et al. (54)	Y	Y	NR	Y	N	Y	Y	NA	Y	Y	Y	NA	N	N	Fair
Kwako et al. (39)	Y	Y	NR	Y	N	Y	Y	Y	Y	Y	Y	NA	N	Y	Good
Heinz et al. (49)	Y	Y	NR	Y	N	NA	NA	Y	Y	NA	Y	NA	NA	N	Poor
Kaczkurkin et al. (53)	Y	Y	NR	Y	N	Y	Y	N	Y	Y	Y	Y	N	N	Good
Ralevski et al. (40)	Y	Y	NR	Y	N	Y	Y	Y	Y	Y	Y	NA	NA	N	Good
McHugh et al. (46)	Y	Y	NR	Y	N	Y	Y	N	Y	N	Y	N	NA	N	Poor
Peck et al. (57)	Y	Y	NR	N	N	Y	Y	NA	Y	N	Y	NA	N	N	Poor
Somohano et al. (37)	Y	Y	NR	Y	N	NA	NA	N	Y	NA	Y	N	Y	N	Poor
Lyons et al. (48)	Y	Y	NR	N	N	NA	NA	NA	Y	NA	Y	NA	NA	Y	Poor
Rosenblum et al. (55)	Y	Y	NR	Y	N	NA	NA	NA	Y	NA	Y	N	NA	N	Poor
Vogel et al. (59)	Y	Y	NR	Y	N	Y	Y	N	Y	Y	Y	NA	N	Y	Good

*Y, Yes; N, No; NR, Not Reported; NA, Not Applicable*.

*Q1: Was the research question or objective in this paper clearly stated?; Q2: Was the study population clearly specified and defined?; Q3: Was the participation rate of eligible persons at least 50%?; Q4: Were all the subjects selected or recruited from the same or similar populations (including the same time period)? Were inclusion and exclusion criteria for being in the study prespecified and applied uniformly to all participants?; Q5: Was a sample size justification, power description, or variance and effect estimates provided?; Q6: For the analyses in this paper, were the exposure(s) of interest measured prior to the outcome(s) being measured?; Q7: Was the timeframe sufficient so that one could reasonably expect to see an association between exposure and outcome if it existed?; Q8: For exposures that can vary in amount or level, did the study examine different levels of the exposure as related to the outcome (e.g., categories of exposure, or exposure measured as continuous variable)?; Q9: Were the exposure measures (independent variables) clearly defined, valid, reliable, and implemented consistently across all study participants?; Q10: Was the exposure(s) assessed more than once over time?; Q11: Were the outcome measures (dependent variables) clearly defined, valid, reliable, and implemented consistently across all study participants?; Q12: Were the outcome assessors blinded to the exposure status of participants?; Q13:Was loss to follow-up after baseline 20% or less?; Q14: Were key potential confounding variables measured and adjusted statistically for their impact on the relationship between exposure(s) and outcome(s)?*.

### Study Results

#### Study Characteristrics

Twenty-seven studies fulfilled criteria for inclusion in this review, of which 12 focused on alcohol, 4 on tobacco, 1 on cannabis, 1 on cocaine, and 9 on various substance use disorders (three studies on AUD and/or Cocaine Use Disorder and six studies on different types of SUD). Among the 27 included studies, 13 were experimental studies, 13 were observational studies and 1 was a randomized controlled trial.

In total, 3580 subjects were enrolled, of which 1960 (54.7%) met criteria for PTSD and SUD, 1206 (33.7%) for SUD only, and 105 for PTSD only. One study (37) did not indicate the prevalence of low vs. high PTSD scores. Participants were most often males (65.6%), with a mean age of 41.3 years. Most participants (*n* = 3,337; 93.2%) were recruited in care facilities including outpatient (*n* = 1804; 54%), inpatient (*n* = 497; 15%), residential (*n* = 497; 15%) or either inpatient/outpatient (*n* = 539, 16%) treatment programs. Among the included participants, 61.8% (*n* = 2212) met criteria for AUD, 10.9% (*n* = 390) were current smokers, 10.7% (*n* = 383) met criteria for Cocaine Use Disorder, 9.6% (*n* = 343) for Cannabis Use Disorder, 6.7% (*n* = 241) for Opiate Use Disorder, 3.3% (*n* = 118) for Stimulant Use Disorder, 2.4% (*n* = 85) for Anxiolytic or Hypnotic Use Disorder, 2.2% (*n* = 79) for comorbid Cocaine and Alcohol Use Disorder, and 1.2% (*n* = 45) for Polysubstance Use Disorder.

A detailed description of all studies included and their main results can be found in Tables 3, 4.

**Table 3:** Details of experimental studies included in the review.

**Study**	**Sample**	**Method**	**PTSD evaluation**	**Craving evaluation**	**Results**
**Brady et al**. **(****41****)** (Alcoholism: Clinical and Experimental Research)	63 adults (35 men, 28 women), with AUD alone (*n* = 35) or associated with PTSD (*n* = 28) according to DSM-IV, recruited *via* advertisements during a 36-month period. Non-inclusion criteria: current mood disorder, major somatic disorder, psychotic disorder, behavioral disorder, bipolar disorder, corticoids, antidepressants, anxiolytics, mood regulators, beta blockers on the last month, opiates agonist or antagonist during last 2 weeks, pregnancy, breast feeding or ineffective contraception, other use disorder (except caffeine, nicotine), according to the Structured Clinical Interview for DSM-IV (SCID-IV). AUD diagnosis with SCID-IV. Alcohol use during 30 days before and after test assessed with the Timeline Follow-back (TLFB), urinary, and breath tests.	Experimental non-randomized study. Exposure to a stressor with craving evaluation, stress level, biological stress response (ACTH and cortisol), right before and after test, and after 5, 30, 60, and 120 min. Evaluation of alcohol use 1 week and 1 month after experimental session.	Current PTSD assessed with the Clinician Administered PTSD Scale (CAPS), according to DSM-IV.	Self-evaluation: Within Session Rating Scale: WSRS (craving analog visual scale).	No significant difference between subjects with AUD with and without PTSD concerning craving and stress level before and after exposition. Among subjects with AUD alone, craving and stress level post-exposition are predictive of alcohol use after 1 week and 1 month. Among subjects with AUD and PTSD, only alcohol use before test was predictive of alcohol use post-exposure. Among subjects with PTSD: no correlation between corticotrope axis response to stress and alcohol use.
**Schumacher et al**. **(****42****)** (The American Journal on Addictions)	42 adults (13 males, 29 females) aged 25–56 years, meeting DSM-IV criteria for AUD and PTSD, with a criterion A traumatic event <18 years. Participants were recruited from two addiction treatment programs in the Northeast USA.	Experimental study. Participants were exposed to traumatic script and alcohol-related cues, then craving was assessed. The authors analyzed the correlations between age of first trauma (before 13 years old vs. 13–18 years old), severity of PTSD and AUD, and post-exposure craving.	PTSD diagnosis with CAPS according to DSM-IV. Presence of A PTSD criterion of DSM-IV with National Women's Study (NWS) PTSD module. PTSD severity assessed with the Impact of Event Scale-Revised (IES-R).	Self-evaluation with analog visual scale (0–10)	86% of participants had their first trauma <13 years old, and 71% had their first episode of drunkenness ≥13 years old. There was no correlation between age of first trauma and first drunkenness. Subjects who had experienced trauma in childhood (<13 years), had more severe AUD, PTSD symptoms (specifically intrusive symptoms), and post-exposure craving than subjects who experienced their first trauma later (13–18 years). However, there was no significant difference regarding alcohol consumption in response to negative emotions.
**Coffey et al**. **(****30****)** (Psychology of Addictive Behavior)	43 subjects (67% women) with comorbidity AUD and PTSD according to DSM-IV who had consumed alcohol in the past 60 days. They were recruited in two outpatient addiction treatment centers in New York. Non-inclusion criteria: psychotic disorder, current manic episode, current severe depressive episode, military trauma, exposure therapy. Participants were not excluded if they met criteria for a SUD other than alcohol.	Randomized experimental study. First lab session: exposure to trauma and neutral scripts alone, then exposure to trauma script followed by neutral or alcohol cues. After each exposure, assessment of craving, emotional distress, and negative emotions. Then randomization in a group of exposure therapy in imagination or a group of therapy based on relaxation (six sessions). Second lab session: identical to the first.	PTSD diagnosis with CAPS according to DSM-IV. Presence of A PTSD criterion of DSM-IV with NWS PTSD module. PTSD severity assessed with the IES-R.	Self-evaluation with analog visual scale (0–10)	No difference in PTSD symptom severity (CAPS or IES-R) between the two groups (expo and relaxation) before randomization. Participants had increased alcohol craving and emotional distress after exposure to trauma script and alcohol cues. The exposure therapy group had a decrease in post-exposition alcohol craving and emotional distress between the two lab sessions (unlike relaxation group). Subjects who had experienced alcohol craving in the first lab session and completed the six therapy sessions had non-significant decrease in PTSD symptoms between the two lab sessions.
**Coffey et al**. **(****31****)** (Experimental and Clinical Psychopharmacology)	40 adults (63% women) with AUD and PTSD according to DSM-IV, who used alcohol in last 60 days. Participants were recruited in a residential care clinic. Non-inclusion criteria: psychotic disorder, current manic episode, benzodiazepine use. Another use disorder was not considered as non-inclusion criterion. Alcohol use disorder screening with the Alcohol Use Disorder Identification Test (AUDIT), and diagnosis with Computerized Diagnostic Interview Schedule. (C-DIS IV). Symptoms and use consequences assessed with Alcohol Dependence Scale (ADS). Objective use measures with urinary and breath tests.	Experimental non-controlled study, non-randomized. Subjects are exposed to four exposition combinations (script related to traumatic events, alcohol cues, neutral cues) Measures after each combination: •Salivary flow •Craving •Emotional distress •Arousal state	PTSD diagnosis with CAPS according to DSM-IV. Presence of A PTSD criterion of DSM-IV with NWS PTSD module. Evaluation of PTSD symptom severity with IES-R.	Self-evaluation with analog visual scale from 0 to 10	Exposure to traumatic script and to alcohol cues led to significantly superior responses (more craving, emotional distress, salivation, and arousal), from neutral expositions. The association between trauma script exposure following with alcohol cue was associated with the more intense craving level.
**Nosen et al**. **(****38****)** (Behavior Modification)	108 adults (58 men and 50 women) with DSM-IV criteria for AUD and PTSD and with at least one day of massive use during last 60 days. Subjects were recruited in a community addiction care center. Non-inclusion criteria: psychotic disorder, current manic episode, benzodiazepine use, or any other medication that could affect craving or salivation. Another use disorder was not considered as non-inclusion criterion. Use measures were identical to Coffey et al study.	Similar method as Coffey et al. with exposition combination, and measures after each combination: •Salivary flow •Craving •Positive and negative affects	Identical measures from Coffey et al.	Self-evaluation with 3 Likert Scales (0–10)	Combination of traumatic script followed by alcohol cue exposure led to more intense craving. Adding alcohol cue to neutral script or traumatic script increased significatively positive and negative affect. In non-traumatic conditions (neutral script), craving level was correlated with negative and positive affect intensity aroused by alcohol cue. In traumatic conditions (traumatic script), only negative affect intensity provoked by alcohol cue was correlated with craving level. Participants classified as ≪ ambivalent ≫ responders to alcohol related cues (high rise of positive and negative affect) reported the most intense craving.
**Kwako et al**. **(****39****)** (Addiction Biology)	52 subjects (55% male) aged 21–50 years, with comorbid AUD and PTSD according to DSM-IV criteria, participating in a study of the efficacy of NK1 antagonists in comorbid subjects. Recruitment was done through a newspaper advertisement. SCID-IV was used for diagnosis of AUD. The severity of addiction was measured by the ADS and alcohol consumption was assessed subjectively (TLFB) and objectively (breath test). Finally, the consequences of alcohol consumption were investigated with the Addiction Severity Score (ASI). Non-inclusion criteria: severe medical problems, inability to participate in all study procedures, inability to provide informed consent.	Experimental study. Comparison of two methods of craving induction by stress (Trier test or traumatic script) or by alcohol-related cues, in subjects with PTSD and AUD. Several measures were performed before and after each test: •Anxiety (SUDS, State Trait Inventory Anxiety: STAI). •Craving (Alcohol Urge Questionnaire: AUQ). •Serum cortisol and ACTH.	Current PTSD diagnosed with the SCID-IV, according to DSM-IV. Severity of PTSD symptoms assessed with the PTSD Symptom Severity Index (PSSI). Childhood Traumatic Event Search with the Childhood Trauma Questionnaire (CTQ).	Self-evaluation with the AUQ.	Both the traumatic and alcohol-related scripts induced significantly higher craving for alcohol than the neutral script. The peak craving induced by exposure to the traumatic script was significantly greater than that induced by the Trier test, which was itself greater than that obtained after exposure to a neutral script. There was no correlation between craving intensity and endocrine response (ACTH and cortisol) after the different tests. Anxiety's level following the Trier Test and the exposure to the traumatic script was significantly higher than the alcohol-related and neutral exposures.
**Ralevski et al**. **(****40****)** (Alcoholism: Clinical and Experimental Research)	25 subjects (92% male) aged 21–65 with comorbid AUD and PTSD according to DSM-IV. Data came from a 12-week double-blind randomized trial comparing Prazosin vs. placebo. Subjects were required to have ≥1 day of heavy drinking (five standard drinks for men, four drinks for women) in the past 14 days. Diagnosis of AUD by SCID, assessment of alcohol consumption over the last 90 days by TLFB. Non-inclusion criteria: pregnant and lactating women, bipolar disorder, schizophrenic disorder, treatment for AUD, suicidal ideation, health problems contraindicating Prazosin. Another use disorder was not considered as non-inclusion criteria.	Experimental study. Exposure to three scripts (traumatic, non-specific stress, and neutral), with several measures taken before and after each exposure: •Craving •Level of anxiety •Negative and positive affect •Heart rate, blood pressure •Salivary cortisol	Diagnosis of current PTSD by the SCID-IV according to DSM-IV. Severity of PTSD assessed by the CAPS	Self-evaluation by the AUQ	Craving, heart rate and blood pressure measured after exposure to the traumatic script were significantly higher than the other two exposures. The level of anxiety and negative affect after exposure to the traumatic and non-specific stress scripts were higher than the neutral script. Craving intensity after exposure to the trauma script only, was correlated with the number of heavy drinking days prior to the study. There was no correlation between the intensity of craving induced by the different scripts and the severity score of traumatic symptoms measured by the CAPS.
**Beckham et al**. **(****43****)** (Addictive Behaviors)	129 smoking adults with (*n* = 82) and without (*n* = 47) PTSD, smoking at least 10 cigarettes a day, recruited by advertisements. Non-inclusion criteria: other use disorder, psychotic disorder, bipolar disorder. Addiction severity assessed with Fagerström questionnaire.	Experimental study. Participants were exposed randomly to one of three types of personalized scripts (traumatic, stressful, neutral), then received randomly two types of cigarettes (with and without nicotine). Several evaluations were repeated at 0, 20, 35, and 50 min: •PTSD symptoms •Negative affect •Craving	PTSD diagnosis with the CAPS according to DSM-IV. PTSD symptoms severity assessed with the Davidson Trauma Scale (DTS).	Self-evaluation with Questionnaire of Smoking Urges (QSU)	Expositions to traumatic scripts and to a lesser extent stressful script led to significant craving, negative affect, and traumatic symptoms severity increase. Effects were more important among smoking patients with PTSD. Smoking, whatever cigarette type, led to craving decrease (significatively more for cigarettes with nicotine), negative affect and traumatic symptoms after exposure to traumatic and stressful scripts, in groups with and without PTSD.
**Dedert et al**. **(****44****)** (Nicotine and Tobacco Research)	47 smokers (68% men) smoking at least 15 cigarettes a day, with expired carbon monoxide: CO ≥ 15 ppm, with (*n* = 17) or without (*n* = 30) associated PTSD, who were not seeking treatment for nicotine dependence. Subjects were recruited *via* flyers and clinician referrals from local outpatient clinics. Non-inclusion criteria: instable somatic disease, acoustical deficit, smoking tobacco in another form than cigarettes, benzodiazepines use, psychotic disorder, manic or depressive current episode, past PTSD, other substance use disorder. Addiction severity was assessed with Fagerström questionnaire.	Experimental study: participants were assigned randomly to one of the three groups (regular cigarette, low nicotine cigarette, no cigarette), then exposed to a neutral script. Subjects fulfilled several evaluations before and after expositions: •Withdrawal symptoms •Craving level •Traumatic symptoms	PTSD Diagnosis with CAPS according to DSM-IV. PTSD symptoms severity assessed with DTS.	Self-evaluation of craving with QSU-Brief. Craving was also assessed with withdrawal symptoms using Shiffman/Jarvik Withdrawal Scale.	PTSD diagnosis or traumatic symptoms severity did not influence initial smoking level. After one night abstinence, subjects with PTSD had more craving and behavioral withdrawal symptoms. They smoked in anticipation of pleasure and of a decrease in negative affect. After smoking, PTSD subjects had less craving release sensation. Among subjects in ≪ non-cigarette ≫ group, those with PTSD reported increase of negative affect between two measures, contrary to subjects without PTSD.
**Tull et al**. **(****45****)** (Journal of Experimental Psychopathology)	60 subjects (55% male) aged 20–58, with cocaine use disorder according to DSM-IV and a history of trauma exposure, admitted for treatment in a residential addiction treatment facility. Subjects were required to have a Mini-Mental State Examination (MMSE) score ≥ 24. Diagnosis of cocaine use disorder made by the SCID-IV, severity of cocaine use over the past year by a Likert scale (0–5). Non-inclusion criteria: current psychotic disorder (determined by SCID-IV).	Experimental study. Participants were exposed to traumatic and neutral scripts with pre- and post-test measures: •Negative affect •Craving	Current PTSD diagnosed with CAPS according to DSM-IV	Self-evaluation with Likert Scale (0–10)	After exposure to the traumatic (but not neutral) script, subjects with PTSD had significantly higher craving for cocaine than other participants. In male subjects only, negative affect (shame, guilt) mediated the relationship between traumatic symptoms and cocaine craving.
**Coffey et al**. **(****32****)** (Drug and Alcohol Dépendance)	75 participants: 30 subjects (87% women) with PTSD + cocaine use disorder according to DSM-IV and 45 subjects (46% women) with PTSD + AUD according to DSM-IV. All had used alcohol and/or cocaine at least once in the last 60 days. Participants were recruited from outpatient or inpatient treatment programs at the Medical University of South Carolina and local treatment facilities in the Charleston area. Non-inclusion criteria: psychotic disorder, current manic or severe depressive episode, current PTSD treatment. Subjects with other SUD were not excluded. Subjects with AUD could also have cocaine use disorder and vice versa.	Experimental study. First session: assessment of PTSD, SUD, consequences of consumption, then creation of trauma script. Second session: assessment of initial craving, then exposure to four combinations (Trauma script + substance cue: TD, Trauma script + wood chips: TN, neutral script + substance cue: ND and neutral script + wood chips: NN). Then assessment of craving and emotions.	PTSD diagnosis with the CAPS and NWS PTSD Module, according to DSM-IV.	Self-evaluation with the Cocaine Craving Questionnaire (CCQ) for cocaine, Alcohol Craving Questionnaire (ACQ) for alcohol and analog visual scale for both.	No difference between alcohol and cocaine use disorder groups regarding PTSD and depressive symptoms. For all participants, initial craving was not correlated with post-exposure craving. Craving, and negative affect after TD and TN exposures was higher than after ND and NN exposures. Post-exposure craving in AUD group was higher than cocaine disorder group.
**Saladin et al**. **(****47****)** (Addictive Behaviors)	124 subjects among which 70 had AUD, 54 crack use disorder according to DSM-IV criteria, who used during last 60 days. Subjects were recruited in addictology service in Medical University of South Carolina or in regional addictology center. Every participant must have suffered from physical and/or sexual violence concordant with DSM-IV A PTSD criterion. 61% of the sample (*n* = 76) met criteria for current PTSD Non-inclusion criteria: psychotic disorder, current manic or depressive episode. Other use disorders were not considered as non-inclusion criteria.	Experimental non-controlled, non-randomized study. Subjects were exposed to traumatic, alcohol, crack, or neutral cues. After each exposition, subjects reported their craving level.	PTSD diagnosis with CAPS according to DSM-IV. 66% subjects with AUD and 56% with crack use disorder had PTSD (61% of total sample). Self-evaluation of traumatic severity symptoms with IES-R.	Self-evaluation with analog visual scale in 21 points	Exposition to traumatic script or substance cue led to craving significatively superior from exposition to neutral scripts and cues. During traumatic script exposition, craving level was positively correlated with PTSD symptom severity, independently from substance exposition. During exposition to substance cue, craving severity was not correlated with traumatic symptoms, only if this exposition was preceded with traumatic script exposition.
**McHugh et al**. **(****46****)** (Comprehensive Psychiatry)	194 adults (50% women) aged 18 to 65, with a history of traumatic exposure according to criterion A PTSD in DSM-IV (27.3% had PTSD), and a current alcohol and/or cocaine use disorder according to DSM-IV. They were recruited in a residential addiction treatment center. 26.3% had cocaine use disorder, 33% had alcohol use disorder and 40.7% had both. Non-inclusion criteria: cognitive impairment (MMSE < 24)/psychotic disorder.	Experimental study. First session: assessment of PTSD (CAPS), SUD (SCID-IV), and anxiety sensitivity (Anxiety Sensitivity Index-3: ASI-3). Second and third sessions: exposure to traumatic and neutral scripts. Measurement of negative affect and craving before and after exposure. Analysis of the correlations between anxiety sensitivity, PTSD severity, negative affect, and craving.	PTSD diagnosis with the CAPS according to DSM-IV	Self-evaluation with a Likert Scale (0–10).	There was a positive correlation between anxiety sensitivity and PTSD severity. Subjects with PTSD had higher anxiety sensitivity. Traumatic exposure resulted in increased craving and negative affect. Anxiety sensitivity was positively correlated with post-traumatic exposure negative affect but not with craving. PTSD severity was positively correlated with post-traumatic exposure negative affect and craving.

**Table 4:** Details of observational and interventional studies included in the review.

**Study**	**Sample**	**Method**	**PTSD evaluation**	**Craving evaluation**	**Results**
**Drapkin et al**. **(****51****)** (Journal of Substance Abuse Treatment)	512 subjects aged 19–81 years: 167 subjects seeking care for comorbid AUD and PTSD; 105 subjects seeking care for PTSD alone; and 240 subjects seeking care for AUD alone. The three groups came from three randomized controlled trials. Recruitment was made in general population through advertisements in local newspapers. Non-inclusion criteria: bipolar or psychotic disorder. Participants in AUD group could not have PTSD diagnosis, participants in PTSD group could not have AUD, contraindication to sertraline or previous failure to Sertraline. Subjects with AUD could not have Naltrexone contraindication or other SUD diagnosis.	Cross-sectional study. Comparison of psychosocial variables between subjects with comorbid AUD and PTSD, and subjects with PTSD or AUD alone.	PTSD diagnosis with the PSS-I and the Structured Interview For PTSD (SIP) according to DSM-IV.	Self-evaluation with the Penn Alcohol Craving Scale (PACS).	Comorbid subjects had less employment, less college education, and lived alone more often than PTSD or AUD group and had lower income than PTSD group. Comorbid subjects were not different in terms of alcohol consumption compared to subjects with AUD alone, but experienced more craving. Comorbid subjects had more depressive symptoms than subjects with PTSD alone.
**Jayawickreme et al**. **(****50****)** (Psychology of Addictive Behavior)	167 subjects (34% female) seeking care for comorbid PTSD and AUD according to DSM-IV criteria. Participants were recruited through advertisements in the University of Pennsylvania's Center for the Treatment and Study of Anxiety. Non-inclusion criteria: SUD other than alcohol, tobacco, cannabis, bipolar and psychotic disorders, opiate use in past month, somatic problems that may interfere with addiction treatment, pregnancy or risk of pregnancy.	Cross-sectional study. Analysis of correlations between sex, traumatic cognitions, craving, and addiction consequences.	PTSD diagnosis with the PSS-I. Posttraumatic cognitions assessed with the Posttraumatic Cognitions Inventory (PTCI).	Self-evaluation with the PACS.	In men only, traumatic cognitions (specifically self-depreciation) were correlated with craving. Traumatic cognitions (self-deprecation and guilt) were correlated with negative consequences related to addiction.
**Simpson et al**. **(****52****)** (Psychology of Addictive Behaviors)	29 subjects (93% men) with AUD according to DSM-IV criteria, who used alcohol during last 30 days. 89, 7% of participants had PTSD. Subjects were recruited in a veteran medical center (*n* = 24) or in an urban addiction center in Seattle (*n* = 5). Another use disorder was not considered as non-inclusion criteria. Use severity was assessed with the AUDIT.	Evaluation of interactions between traumatic symptoms and craving. Observational study in everyday life during 28 days. Subjects reported everyday PTSD symptoms severity and their craving level using Interactive Voice Response (IVR).	PTSD diagnosed with the PTSD Check List (PCL-C) according to DSM-IV.	Self-evaluation with the PACS.	Initial PTSD severity was correlated with craving and alcohol use. Traumatic symptom severity (irritability and outbursts) was correlated with craving levels on the same day. Traumatic nightmares, affective blunting and hypervigilance symptoms were correlated with craving levels the next day. However, there was no correlation between craving level sand traumatic symptom severity the next day.
**Heinz et al**. **(****49****)** (Military Medicine)	68 military veterans (90% male) with AUD according to DSM-IV, wanting to stop or reduce their alcohol consumption and having been exposed to a traumatic event during their life. The participants came from a randomized controlled trial concerning Topiramate. They were recruited from the San Francisco Veterans Affairs Medical Center. Diagnosis of AUD with SCID-IV. Alcohol use in the past 90 days was assessed with the TLFB. Non-inclusion criteria: unstable psychiatric or somatic disorder, suicidal ideation or suicide attempt in the last 6 months.	Cross-sectional observational study. Assessment of cognitive functions (processing speed, executive functions, risk-taking/impulsivity, verbal learning, and memory), and analysis of correlations with different variables (alcohol consumption, craving, and severity of PTSD symptoms).	PTSD diagnosed with the PCL-C according to DSM-IV.	Self-evaluation with the Obsessive Compulsive Drinking Scale (OCDS).	Severity of PTSD symptoms was positively correlated with craving and alcohol consumption in the past 3 months. Craving intensity was also correlated with the frequency and quantity of alcohol consumption. Lower verbal learning and memory were correlated with more intense alcohol consumption. Higher level of impulsivity was correlated with stronger craving.
**Kaczkurkin et al. (2016)** (Behavior Research and Therapy)	165 subjects (65.5% male) seeking care for comorbid PTSD and AUD according to DSM-IV-TR. Recruitment at the University of Pennsylvania's Center for the treatment and Anxiety Studies and the Philadelphia Veteran's Affairs Hospital. Inclusion criteria: current diagnoses of PTSD and AUD according to DSM-IV-TR criteria, PSS-I score ≥ 15, consumption > 12 drinks/week in the past month, with 1 day with ≥ 4 drinks consumed. Non-inclusion criteria: SUD other than alcohol, tobacco, or cannabis, current psychotic disorder; suicidal ideation or, opioid use during the last month, diseases that may interfere with treatment, pregnancy, or nursing.	Randomized controlled trial. The participants were randomized to four treatment groups (Naltrexone + Prolonged exposure, Naltrexone alone, placebo + Prolonged exposure, placebo alone). Different measures were collected every four weeks, before, during and after the treatment (PTSD symptoms, percentage days drinking, craving).	Current PTSD diagnosis with PSS-I according to DSM-IV.	Self-evaluation with the PACS.	**Baseline measures:** PTSD severity was positively correlated with the percentage of drinking days and craving. The % of drinking days was positively correlated with alcohol craving. **Evolution with treatment:** The Naltrexone + prolonged exposure group had significantly greater reduction in craving than the Naltrexone alone group. There was no difference in terms of reduction in % drinking days between groups. Subjects with higher initial PSS-I score had a more rapid decrease in craving over time. Craving at a given time was correlated with measures of % drinking days and severity of traumatic symptoms at previous time.
**Lyons et al**. **(****48****)** (Journal of Dual Diagnosis)	136 veterans (90% male), with full or subthreshold (one missing symptom) PTSD, and alcohol abuse or dependence, seeking treatment, enrolled in a randomized controlled trial. The participants had at least 20 days of massive use (four drinks for females and five drinks for males) over the past 90 days. Alcohol consumption over the past 90 days was assessed by the TLFB.	Cross-sectional observational study, using data collected from a randomized controlled trial. The study assessed the relationship between PTSD negative cognitions, negative affect, and alcohol craving.	PTSD diagnosis with the CAPS according to DSM-5.	Self-evaluation with PACS.	Alcohol craving was positively correlated with the number of massive drinking days, severity of PTSD symptoms, negative affect, and trauma-related cognitions. Negative emotions mediated relationship between trauma-related cognitions and alcohol craving.
**Dedert et al**. **(****44****)** (Nicotine and Tobacco Research)	52 adult smokers (18–65 year olds) with PTSD who smoked at least 10 cigarettes a day. Recruitment took place in the general population or in a veteran medical care center using flyers or brochure from previous study. Non-inclusion criteria: nicotine use in another form than cigarettes, instable major somatic disease, use of bupropion or benzodiazepines. Another use disorder was not considered as non-inclusion criteria. Addiction severity was assessed using Fagerström questionnaire.	Observational everyday life study using EMA method. Subjects were followed over 2 weeks (1 week smoking freely then 1 week after stopping smoking). Participants had to fulfill evaluations randomly during the day (every 2–3 h first week, then 1–2 h second week). Those evaluations included: •PTSD symptoms •Negative affect •Craving	PTSD diagnosis using CAPS according to DSM-IV PTSD symptoms severity assessed with DTS	Self-evaluation of craving with a scale (1–5)	Compared to free use period, abstinence period was marked by decrease of PTSD and mean craving levels, but not negative affect. Variability of traumatic symptoms from one measure to another and negative affect decreased during abstinence period. Avoidance symptoms, hypervigilance and negative affect during EMA evaluation were correlated with craving level during next EMA evaluation, but not the reverse.
**Rosenblum et al**. **(****55****)** (Journal of Dual Diagnosis)	162 US Army veterans (6.4% female) aged 18–65 years, smoking at least 10 cigarettes a day, with expired CO ≥ 8 ppm, with or without PTSD or current depressive episode. Subjects were recruited by flyers from a veterans affairs hospital in Wisconsin. Addiction severity assessed with Fagerstrom and motivation to smoke with the Brief Wisconsin Inventory For Smocking Dépendance Motives (WISDM). Non-inclusion criteria: other psychiatric disorders, use of Varenicline or Bupropion.	Cross-sectional observational study assessing motivational processes influencing tobacco addiction in smokers with PTSD or depressive episode.	Current PTSD diagnosis using CAPS according to DSM-IV.	Self-evaluation with the Brief WISDM.	Subjects with PTSD had higher mean Fagerstrom scores than other participants. Subjects with PTSD or depressive episode had greater craving than the control group. No significant difference in craving was found between subjects with PTSD and depressive episode.
**Boden et al**. **(****56****)** (The American Journal on Addictions)	94 US military veterans (94% men), with cannabis use disorder according to DSM-IV-TR (with DSM 5 withdrawal criteria), asking for care. Subjects were recruited using advertisements in an outpatient PTSD clinic among veterans during previous study. Non-inclusion criteria: intellectual deficit, already have decreased daily cannabis use from at least 25% during last month, pregnancy, breast feeding, suicidal thoughts. Another use disorder was not considered as non-inclusion criterion. Tobacco, alcohol, and cannabis use were measured using TLFB, cannabis use motives with the Marijuana Motives Measure (MMM), use consequences with the Marijuana Problems Scale (MPS), and cannabis withdrawal symptoms with the Marijuana Withdrawal Checklist (MWC).	Cross-sectional observational study comparing subjects with cannabis use disorder with and without PTSD on their cannabis use and its consequences.	PTSD diagnosis using CAPS according to DSM-IV, Self-evaluation of traumatic symptoms severity with PCL-M	Self-evaluation with the Marijuana Craving Questionnaire (MCQ)	Subjects with PTSD used more often cannabis as coping strategy. Subjects with PTSD experienced a higher craving level during compulsive, emotional and anticipatory use. Traumatic symptom severity was positively correlated with withdrawal symptom intensity and emotional component of craving.
**Driessen et al**. **(****58****)** (Alcoholism: Clinical and Experimental Research)	459 subjects aged 15–60 years, treated in addictology in Germany (73% inpatients, 10% followed in a day clinic, 17% outpatients) for a SUD (alcohol: 66% and/or other substance 60%). Participants had to be abstinent from all psychoactive substances for at least 2 weeks. Diagnosis of SUD with International Diagnostic Checklist (IDCL) according to DSM-IV, assessment of addiction severity with the ASI, objective measurement of substance use with urine and breath tests.	Cross-sectional observational study. Analysis of the relationship between PTSD diagnosis, type of addiction (alcohol or other substances), addiction severity, and craving intensity. Participants were classified by addiction type: •Group A (AUD alone; *n* = 182) •Group D (SUD other than alcohol; *n* = 154) •Group AD (AUD + SUD; *n* = 123) And by PTSD status: •PTSD group (score positive on the IDCL and PDS) •Subthreshold PTSD group (score positive on the IDCL or PDS) •Subjects who have been exposed to a traumatic event without PTSD •Subjects who have never been exposed to a traumatic event.	Diagnosis of current PTSD according to DSM-IV by IDCL and Posttraumatic Diagnostic Scale (PDS).	Assessed with ASI	Prevalence of PTSD in groups AD and D was significantly higher than group A. Subjects with PTSD had a higher addiction severity score on ASI, a greater number of inpatient admissions to addiction care, a shorter mean time of abstinence between relapses, and experienced more frequent craving than other participants. Addiction severity was higher in subjects with a SUD other than alcohol.
**Wieferink et al**. **(****33****)** (Addictive Behaviors)	297 Dutch subjects, aged 17–73 years (72% male) followed between 2012 and 2014 for SUD according to DSM-IV criteria. Assessment of past 30 days and lifetime substance use by the MATE substance use Inventory. Non-inclusion criteria: severe psychiatric or somatic disorder.	Prospective study, assessing efficacy of standard, non-integrated SUD treatment in subjects with higher or lower PTSD symptoms.	Diagnosis of current PTSD by SRIP according to DSM-IV.	Self-evaluation with the OCDS.	**Baseline assessment:** Subjects with high severity of PTSD symptoms had significantly higher levels of craving and anxiety-depressive symptoms than other participants. However, there was no significant difference in the number of days of use. **Measures at 3 and 6 months** **of treatment:** Subjects had a significant decrease in days of use regardless of PTSD symptom severity. Subjects with high severity of traumatic symptoms had a significantly greater decrease in craving.
**Peck et al**. **(****57****)** (Journal of Anxiety Disorders)	72 military veterans (71 men and 1 woman) with PTSD and SUD according to DSM-IV-TR, in treatment in veterans medical center of South-East USA. Subjects were recruited during inscription to cognitive processing therapy program of 6 weeks proposed by the medical center. Use frequency was assessed with the Brief Addiction Monitor (BAM).	Prospective study. Subjects participated in PTSD-specific treatment (cognitive processing therapy) during 6 weeks. Evaluations were performed before and after treatment: •PTSD symptoms •Craving •Traumatic cognitions •Depressive symptoms	PTSD diagnosis with the MINI according to DSM-IV-TR. Self-evaluation of PTSD symptoms with PCL-S. Evaluation of erroneous cognitions linked to PTSD with the PTCI.	Self-evaluation with the Craving Questionnaire—Short Form Revised (CQSFR)	Traumatic initial dysfunctional cognitions were correlated with initial craving level. There was no correlation between PTSD symptoms severity and craving. The therapy allowed a significant decrease of craving, PTSD symptoms, depressive symptoms and erroneous traumatic cognitions. Improve of dysfunctional traumatic cognitions with the cognitive processing therapy did not explain craving decrease following the therapy.
**Somohano et al**. **(****37****)** (Journal of Dual Diagnosis)	257 adults with SUD who were abstinent after outpatient or inpatient treatment, waiting for rehabilitation care as part of a randomized controlled trial from 2014. Recruitment was performed with advertisements in addictology treatment centers. Non-inclusion criteria: dementia syndrome, psychotic disorder, suicidal thoughts, subjects who already benefited mindfulness therapy. Use disorder severity was assessed with the Severity of Dependence Scale (SDS).	Cross-sectional observational study, using from data collected previously in a randomized controlled trial during rehab treatment. Evaluation of relationship between PTSD symptoms and craving according to the substance (alcohol, psycho stimulants, opiates, cannabis).	Diagnosis with the PCL-C according to the DSM-IV-TR.	Self-evaluation with PACS adapted for other substances.	For alcohol (*n* = 131), craving was correlated with global PTSD severity, more specifically with hypervigilance symptoms. Concerning psychostimulants (*n* = 66), craving was correlated with global PTSD severity, more specifically with avoidance syndrome. Concerning opiates (*n* = 36): craving was correlated with global PTSD severity, but not with specific PTSD symptoms. Concerning cannabis (*n* = 24), there was no correlation between craving and PTSD.
**Vogel et al**. **(****59****)** (Drug and Alcohol Dependence)	108 adults (25% female) with SUD and comorbid PTSD, beginning inpatient detoxification. Non-inclusion criteria: severe cognitive deficit, symptoms of psychotic disorder.	Observational study, Evaluation of correlation between PTSD symptoms and craving level.	PTSD assessed with the PCL-5 according to the DSM-5. Screening for life trauma event with the Life Events Checklist (LEC-5).	Self-evaluation with the Mannheimer Craving Scale (MaCs)	PTSD symptoms were positively associated with craving level but were not predictors of craving level the following day.

#### Effects of Traumatic Cue- and Stress-Exposure on Craving Across SUD Subgroups

The 13 experimental studies selected for this review consisted, for most part, of exposing participants with comorbid SUD and PTSD to traumatic memories, non-specific stressors, and substance-related cues, and then evaluating their responses across SUD subgroups. Seven experimental studies involved AUD, two involved tobacco use disorder, one involved cocaine use disorder, and three studies included patients suffering from AUD and Cocaine Use Disorder.

##### Alcohol Use Disorder

The primary finding was that exposure to a traumatic memory (in the form of a script recounting a traumatic life event) generated a significantly greater increase in craving than neutral exposure and similarly to exposure to an alcohol-related cue (31, 38–40). The studies by Coffey et al. (31) and Nosen et al.'s (38) went further, showing that the combination of exposure to a traumatic script followed by an alcohol-related cue generated greater craving than each type of exposure when considered separately. Two studies showed that exposure to a traumatic memory increased craving more than a non-specific stressor (39, 40).

Only one study compared subjects with the comorbidity AUD and PTSD to subjects with AUD alone (41). This study found no significant difference between the two groups in terms of craving intensity after exposure to a non-specific stressor. Ralevski et al. (40) found no correlation between the intensity of craving provoked by the different scripts (traumatic, non-specific stress, and neutral) and the severity of PTSD symptoms, measured by the Clinician Administered PTSD Scale (CAPS). Finally, Schumacher et al. (42) showed that subjects who had suffered early childhood trauma (<13 years of age) presented a more severe AUD, traumatic intrusion symptoms, and post-exposure craving (traumatic script and alcohol-related cues), than subjects who suffered their first trauma later in adolescence (13–18 years of age).

##### Tobacco Use Disorder

Two experimental studies addressed tobacco use disorder. Beckham et al. (43) showed that the increase in craving, negative affect and traumatic symptoms were stronger after being exposed to traumatic scripts than stressful event scripts and neutral scripts. Subjects with PTSD experienced a more significant increase in craving and negative affect compared to the other group. Cigarette use was associated with a reduction in craving (which was greater in magnitude after a traumatic script), as well as reduction in negative affect, and traumatic symptoms, independently of group type. Dedert et al. (44) found that subjects with PTSD presented more severe withdrawal symptoms and a higher craving level for two dimensions (anticipation of reinforcing effect, anticipation of withdrawal symptoms and negative affect release) during acute withdrawal. Participants with PTSD reported lower craving reductions after smoking.

##### Cocaine Use Disorder

One experimental study by Tull et al. (45) assessed the effect of exposure to a personalized trauma cue on cocaine craving in patients with cocaine use disorder with or without PTSD. Subjects with PTSD had significantly higher craving for cocaine than other participants after the traumatic script.

##### Multiple Type of Substance Use Disorder: Alcohol and/or Cocaine Use Disorder

Three experimental studies exposed subjects with alcohol and/or cocaine addiction and a history of traumatic events to combinations of traumatic or neutral scripts and substance-related or neutral cues (32, 46, 47). All studies showed that exposure to traumatic memories and substance-related cues increased craving and negative affect significantly more than neutral exposures. The studies of Saladin et al. (47) and McHugh et al. (46) showed a positive correlation between the severity of PTSD symptoms and the intensity of craving after traumatic exposure. Finally, McHugh et al. (46) observed a positive correlation between the level of anxiety sensitivity (tendency to react with fear to signs and symptoms of anxiety) and the severity of traumatic symptoms and negative affect, but not craving after the exposures.

#### Association Between PTSD Symptoms and Craving Across SUD Subgroups

A total of 14 studies investigated the association between PTSD symptoms and craving across SUD subgroups: 12 were observational studies (five for AUD, two for Tobacco Use Disorder, one for Cannabis Use Disorder, and four for different types of Substance Use Disorders), one was an experimental study and one was a Randomized Controlled Trial among AUD individuals.

##### Alcohol Use Disorder

Five cross-sectional observational studies examined correlations between different variables related to PTSD and AUD. The studies by Lyons et al. (48) and Heinz et al. (49) found a correlation between the intensity of craving for alcohol on the one hand and the severity of PTSD symptoms and consumption on the other, in comorbid subjects. For Lyons et al. (48), traumatic cognitions (self-deprecation, dangerousness of the world) generated negative affect, which in turn triggered craving. Jayawickreme et al. (50) also found a positive correlation between traumatic cognitions about oneself (self-depreciation and tendency to blame oneself), negative beliefs about the world and the intensity of craving, but this relationship was only significant in men. Finally, Drapkin et al. (51) compared the psychosocial functioning of subjects with comorbid PSTD and AUD with those with SUD or PTSD alone. The authors found that social functioning was more impaired (less education, lower income, more unemployment), as well as more severe depressive symptoms and cravings, in the comorbid subjects.

In a 28-day study using a daily monitoring with an Interactive Voice Response (IVR), Simpson et al. (52) found that PTSD severity was positively correlated with craving level on the same day but not the following day. In a more specific way, some traumatic symptoms (startle, irritability), were positively correlated with craving levels on the same day, whereas other symptoms (nightmares, emotional blunt, hypervigilance), predicted craving increases on the following day. On the other hand, craving intensity on a given day was not correlated with PTSD symptom severity on the following day.

Two studies assessed the impact of changes of PTSD symptoms overtime on craving after specific treatment approaches. In a randomized clinical trial conducted by Kaczkurkin et al. (53), 165 comorbid subjects were randomly assigned to four different treatment groups: Naltrexone + exposure therapy, Naltrexone alone, exposure therapy + placebo, and placebo alone. At baseline, participants with greater levels of PTSD symptom severity endorsed a significantly greater percentage of days drinking and alcohol craving. The percentage of days drinking was positively correlated with alcohol craving. Participants in the Naltrexone + exposure therapy group had a greater decrease in craving than in the Naltrexone alone group. Subjects with higher initial PTSD severity had a more rapid decrease in craving intensity over time. Finally, the percentage of drinking days and the severity of traumatic symptoms at time t predicted the intensity of craving at time *t* + 1, with alcohol craving being dependent on the amount of PTSD symptoms and alcohol use reported at the previous time point. Coffey et al. (30) compared the effectiveness of exposure therapy and relaxation-based therapy in 43 subjects with comorbid AUD and PTSD using a laboratory-based experiment. A first session conducted before the beginning of treatment showed an increase in craving and emotional distress of the participants after exposure to a traumatic script and alcohol-related cues. Follow-up analyses after six sessions of group exposure therapy found a reduction of both PTSD symptoms and alcohol craving overtime.

##### Tobacco Use Disorder

In 2014, Dedert et al. (54) used Ecological Momentary Assessment (EMA) to follow smokers with PTSD in daily life over 14 days. Participants were allowed to smoke freely during the first week, then had to begin withdrawal without any substitute or pharmacological treatment in the second week. Compared with the pre-withdrawal phase, abstinence was associated with reductions in PTSD symptoms and craving, but not negative affect. During withdrawal period, an increase in traumatic symptom intensity predicted an increase in craving at next EMA evaluation, but the reverse association was not observed.

Rosenblum et al. (55) compared three groups composed by 162 US Army veteran daily smokers: a PTSD group (52 subjects with PTSD alone or with a comorbid depressive episode); a depressive episode group (52 subjects with depressive episode without comorbidity); and a control group (58 subjects with no psychiatric disorder). The PTSD group (with or without depression) described higher craving than the control group without any psychiatric disorder.

##### Cannabis Use Disorder

Boden et al. (56) explored the links between PTSD and different characteristics associated with cannabis use (motivation, relational problems, withdrawal symptoms, and craving) in veterans with cannabis use disorder with and without PTSD. Patients with PTSD used cannabis more frequently as a coping strategy and reported a significatively higher level of craving in several components (compulsive, anticipating release of emotional distress, and use planification). Traumatic symptom severity was positively correlated with the emotional component of craving (release of emotional distress).

##### Multiple Type of Substance Use Disorder

Two studies assessed the course of craving during SUD treatment according to PTSD symptoms severity at baseline. Wieferink et al. (33) assessed outcomes of standard, non-integrated SUD treatment among 297 SUD outpatients (AUD, or Cannabis or Cocaine Use Disorder) with higher (≥48) or lower (<48) PTSD symptom severity based on the Self-Reported Inventory for PTSD (SRIP). At baseline, there was no difference in the number of days of use between subjects, however, subjects with more severe PTSD symptoms had significantly higher levels of craving and anxiety-depressive symptoms. After 3 and 6 months of treatment, there was a decrease in the number of days of use for all subjects, a significantly greater decrease in craving for subjects with more severe traumatic symptoms, and a significant decrease in anxiety-depressive symptoms for subjects with severe traumatic symptoms only. However, patients with higher levels of PTSD symptoms still reported significantly higher scores on depression, anxiety and stress after 6 months of SUD treatment. Peck et al. (57) assessed the impact of a 6 weeks therapeutical program including cognitive processing therapy, Alcoholics Anonymous (AA) meetings, and group discussion with 72 American veterans suffering from PTSD and a substance use disorder (AUD, Cannabis Use Disorder, Cocaine Use Disorder, Opiate Use Disorder, Amphetamine Use Disorder, or Anxiolytic Use Disorder). Baseline dysfunctional cognitions associated with the trauma were positively correlated with PTSD and craving severity. However, PTSD severity was not correlated with craving levels. Cognitive processing therapy was associated with significant improvements in erroneous cognitions, trauma-cued substance craving, and depressive or trauma-related symptoms. Reduction in depressive or trauma-related symptoms was partly explained by the therapy's impact on erroneous cognitions, contrary to craving.

In a cross-sectional study, Driessen et al. (58) focused on the relationship between the type of addiction (alcohol or drug addiction or both), the severity of the addiction and of craving, and the presence or not of comorbid PTSD. Participants with PTSD had a higher addiction severity score, were more often hospitalized, had a shorter abstinence time between relapses and experienced craving more frequently than other participants. Somohano et al. (37) assessed the correlation between severity of different symptoms of PTSD and craving according to four classes of substances: alcohol, psycho-stimulants (cocaine, amphetamines), opiates and cannabis. Concerning subjects with alcohol use disorder (*n* = 131), global PTSD severity and hypervigilance levels were associated with craving intensity. For participants with psychostimulant use disorder (*n* = 66), craving levels were correlated with global PTSD severity and more precisely with avoidance syndrome intensity. Among subjects with opiate use disorder (*n* = 36), global PTSD severity was correlated with craving levels, but with no association to specific symptoms. Finally, for subjects with cannabis use disorder, no variable was associated with craving. An observational study led by Vogel et al. (59), highlighted a positive correlation between craving levels and PTSD symptoms over 6 days following admission for detoxification among comorbid patients (PTSD with alcohol, cannabis, sedatives or mixed use disorder). However, no correlation was found concerning PTSD symptoms at Day 1 and craving the following day.

#### Association of Negative Affect With Trauma Exposure and Craving

##### Alcohol Use Disorder

Several studies focused on the role played by negative affect during different forms of exposures (31, 38, 40). The results were similar to those obtained for craving and showed that exposure to a traumatic script and an alcohol-related cue generated a more intense level of negative affect than during a neutral exposure. Nosen et al. (38) reported that in a traumatic context (exposure to a traumatic script), the intensity of craving was correlated with the severity of negative affect. The study of Coffey et al. (30), through a laboratory-based experiment, found a decrease of both craving and emotional distress after trauma-focused imaginal exposure, suggesting that negative emotions should constitute a mechanism of alcohol craving induced by trauma exposure. Lyons et al. (48) examined more specifically the mediation role of negative affect on the association between PTSD cognitions and craving among 136 treatment-seeking veterans with PTSD and AUD. Mediation models demonstrated that negative affect mediated the association between specific posttraumatic cognitions related to the self, the world, the self-blame, and craving controlling for PTSD/AUD symptom severity and gender. Posttraumatic cognitions were associated with increased negative affect, which in turn was related to increased craving. Finally, one observational study (50) examined sex differences in trauma cognitions and their relationship to symptoms of AUD including craving. Specifically, negative cognitions about the self were associated with increased craving in men, but not in women, a finding that could be related to greater subjective negative emotions related to traumatic experiences in men. In this perspective, higher craving levels could be explained as a result of maladaptive coping of trauma-related negative emotions.

##### Tobacco Use Disorder

The study of Dedert et al. (54) investigated whether craving for cigarettes was driven by PTSD symptoms and negative affect among smokers with PTSD attending to quit, using an EMA procedure. Negative emotions were identified as predictors of craving during the withdrawal period (54). Increased PTSD symptoms and negative affect predicted an increase in craving at the next EMA evaluation, even on days with low levels of craving, but the reverse association was not observed.

##### Cocaine Use Disorder

In the experimental study of Tull et al. (45), in male subjects only, the experience of negative emotions (shame, guilt) in response to the traumatic script mediated the relationship between traumatic symptoms and craving for cocaine. The experience of self-conscious negative affect in response to the trauma script accounted for the relation between PTSD diagnosis and cocaine craving following trauma script exposure.

## Discussion

Twenty-seven studies fulfilled criteria for inclusion in this review, of which 12 focused on alcohol, 4 on tobacco, 1 on cannabis, 1 on cocaine and 9 on various substance use disorders. The results showed that regardless of substance type, PTSD and SUD dual disorder was associated with more intense craving levels and was characterized by a prospective link between PTSD symptom severity and craving episodes. Exposure to traumatic memories in experimental studies was associated with emotional distress whose severity was correlated with craving intensity (31, 38).

Whatever for alcohol (31, 38), tobacco (43), or cocaine (47), experimental results showed that exposure to traumatic cues among subjects with PTSD and substance use disorder comorbidity triggered craving in the same way as exposition to substance cues. There also was an additive effect of the association of both forms of exposure on craving, a finding that is consistent with literature showing an association between exposure to stress and craving among patients with substance use disorder (26, 60). However, beyond stress exposure, there appears to be a specific effect of traumatic memories on craving. According to the study by Beckham et al. (43), exposition to traumatic cues triggered significatively greater craving compared with exposure to non-traumatic stress cues. This result could explain the lack of difference in craving levels between subjects with and without PTSD, after exposition to a cold pressor task (neutral stress) in the investigation by Brady et al. (41). In this way, persons with these comorbidities are repeatedly exposed to traumatic memories and therefore to more intense craving, which could increase the risk of relapse. Moreover, the study by Boden et al. (56) lends support to this interpretation by highlighting the correlation between traumatic symptom severity and craving intensity. Finally, studies using EMA observed a prospective link in the association between PTSD symptoms and craving, showing notably that craving daily variation was a reaction to traumatic symptoms intensity. Such results are generally supportive of self-medication theory, as aggravation of PTSD symptoms would then trigger greater craving and lead to substance use as a means of assuaging traumatic symptoms.

In line with this interpretation, some studies in this review also highlighted the role of negative affect associated with traumatic exposure in the risk of relapse and thereby indicating that substance use may constitute a coping strategy to deal with negative affect. Experimental studies among subjects with alcohol and tobacco use disorder (31, 38, 43) showed a correlation in evolution of negative affect and craving after exposition to a traumatic factor. Moreover, negative emotions were identified as predictors of craving after exposition to traumatic cues as well as in daily follow-ups during withdrawal (54). This literature has also demonstrated a salient association between PTSD, addiction and negative emotions, and points specifically to the mediation role of negative emotions and the relationship between traumatic symptoms and alcohol use (61). According to Zvolensky et al. (62), smokers experience greater negative affect if they have comorbid PTSD. Individuals with comorbidity would also use emotions to assuage emotional distress, in accordance with the principle of negative reinforcement. This dysphoric state could be explained by a decrease in dopaminergic D2 receptor density in the reward network (ventral striatum) among persons with substance use disorders (63) and a higher number of DAT dopamine transporters in persons with PTSD (64) that are correlated with craving intensity. Perturbations of the stress axis could also be implicated in these dual disorders, as anomalies of the stress response among subjects with substance use disorders is associated with the activation of extra hypothalamic corticotropin-releasing factor (CRF) synthesis, excessively activating the amygdala (the brain structure implicated in emotional reactions such as fear) (65, 66). Such hyperactivity has also been observed among individuals with PTSD (67) and it is associated with the presence of enduring negative emotional states (anxiety, irritability, dysthymia). While the prefrontal cortex has a major impact on emotional regulation by the inhibition of the amygdala (68), SUD and PTSD are both associated with a hypoactivation of this area (69, 70) that could explain the major emotional dysregulation among these cases of dual disorder (71, 72). Thus, a negative emotional state or emotional dysregulation characterized by significant fluctuations in daily life could constitute a clinical feature of these dual disorders leading to greater craving frequency and/or intensity, although this hypothesis requires further investigation.

Another important observation of this review that could further understanding for mechanisms underlying PTSD and craving is the impact of early trauma. Schumacher and colleagues (42) demonstrated that patients with dual disorders and early trauma (<13 y.o.) experienced more severe PTSD symptoms, more craving after exposure, and more severe AUD. This is consistent with previous studies highlighting a link between age that the trauma was experienced and PTSD severity (73). The link with craving intensity could be partly explained by the fact that early trauma leads to deficit in inhibitory control during stress exposure, which might facilitate the use of substances as coping strategy (74). Indeed, deficits in inhibition capacities during adolescence is known to be associated with a greater risk of both substance experimentation and the development of substance use disorders (75).

Several limitations of this systematic review should be considered in interpreting its findings. A first concern is the heterogenous nature of the selected studies. Based on the Quality Assessment Tool for Observational Cohort and Cross-Sectional Studies (S2C), most studies included in this review could be qualified as being of “Good” or “Fair” quality (*n* = 17 or 65%). However, nine studies (35%) were classified as “Poor” quality, and this may partly explain the considerable variation observed in study methods. The majority of investigations classified as “Poor” quality were observational studies while most of the “Good” quality studies were cross-sectional in nature. Moreover, the studies used a large variety of questionnaires to assess SUD, PTSD, and craving. Substance use was mainly reported using self-report questionnaires and only six studies used objective methods of assessment (urine or breath tests). Nevertheless, the majority of experimental studies on cue-reactivity administered single-item instruments, mainly visual analog scale which are considered as an acceptable and valid tool in experimental paradigms, while observational studies used different multi-items questionnaires. The heterogeneity of self-report measures of craving and research design in observational studies could explain some variability in the findings. Finally, few studies addressed sex differences in the analysis, although some results indicated specific relationships between trauma-related cognitions and emotions (self-depreciation and self-blame) and craving among males. More comprehensive analyses are needed to examine the impact of sex (and gender) on the underlying relationship between PTSD and SUD across different SUD subgroups. Despite these limitations, the results strongly underscore the strong relationship between PTSD and substance craving and the necessity to concomitantly treat SUD and PTSD as dual disorder.

Concerning treatment approaches, recent studies assessed several classes of pharmacological agents in the treatment of this dual disorder based on neurobiological mechanisms implicated in both disorders when considered individually (76). Moreover, the positive effect on alcohol use and traumatic symptoms was demonstrated with the association of Disulfiram and Naltrexone in a randomized trial (77), and the use of Desipramine led to an improvement of alcohol use and PTSD symptoms (78). Promising results have also been found with treatments using noradrenergic (Prazosine, Propranolol), GABA and glutamatergic system (Memantine, N-Acetyl-Cysteine, and Topiramate). The results of this review also suggest the importance of improving regulation of negative emotions associated with traumatic memories, and treatments of erroneous or dysfunctional cognitions linked with the traumatism. Furthermore, the potential mediation role of post-traumatic cognitions on negative affect and craving raises the issue to consider posttraumatic cognitions and negative emotions as a salient target for craving reduction. On this issue, several therapies targeting emotion regulation and dysfunctional cognitions linked with traumatism such as Prolonged Exposure and Cognitive Processing Therapy were found to be efficacious for substance use, craving and PTSD symptoms (53, 57, 79). The study of Coffey et al. (30) using trauma-focused exposure therapy led to reductions in negative affect and craving, although the potential link between negative post-traumatic cognitions, negative affect and craving was not specifically assessed. Integrated treatment combining prolonged exposure and naltrexone among individuals with comorbid PTSD and AUD demonstrated better outcomes in terms of alcohol craving compared to exposure alone or naltrexone alone. The necessity of global treatment approaches for comorbid patients, including pharmacological treatment, psychotherapies, and psycho-social treatment has also emphasized (80), but further studies are needed in other SUD populations to generalize these findings and examine the temporal changes of emotion dysregulation, trauma-related emotions such as guilt and shame, on subsequent craving and substance use.

## Conclusions

Findings from the current study further inform our understanding of the synergetic relationship between PTSD and SUDs that lead to craving that is greater than that observed with either disorder alone. PTSD symptoms can act as powerful craving cues with an additive effect when combined with exposure to substance-related cues, thereby constituting a salient risk factor for relapse. The craving elicited by PTSD may differ according to specific PTSD symptoms and the effects of specific forms of substance use, although this possibility requires further investigation. Daily life studies using Ecological Momentary Assessment appear to be particularly adapted to investigating the temporal relationship between different PTSD symptoms, emotional states and the clinical expression of addiction, and hold considerable promise for the development of more personalized interventions in dually-diagnosed individuals. Since the majority of the studies included in our review concern alcohol and tobacco, it would be also interesting to expand this research to other substances as well as to behavioral addictions. Specifically, no studies examined the association between craving and MDMA or psychedelic drugs, that is a major issue in view of novel treatment approaches of PTSD.

Our data therefore challenge our current clinical practice in the treatment of patients suffering from dual diagnosis, and argue for the integration of an additional trauma-focused strategies into addiction facilities, notably including cognitive-behavioral therapies based on prolonged exposure. While all individuals suffering from SUD should be systematically assessed for trauma history and PTSD, the present data suggest that PTSD treatment should not be delayed until abstinence has been achieved. The direct relationship between PTSD symptoms and craving argues for the need of these integrated therapies in the goal of providing the most comprehensive and efficacious treatment possible.

## Data Availability Statement

The original contributions presented in the study are included in the article/supplementary material, further inquiries can be directed to the corresponding author/s.

## Author Contributions

FR, LJ, JS, and MF designed the review, wrote, and reviewed the manuscript. FR and LJ reviewed the abstracts and the papers. FR, LJ, and MF obtained the data from the selected articles. All authors approved the final version.

## Conflict of Interest

The authors declare that the research was conducted in the absence of any commercial or financial relationships that could be construed as a potential conflict of interest.

## Publisher's Note

All claims expressed in this article are solely those of the authors and do not necessarily represent those of their affiliated organizations, or those of the publisher, the editors and the reviewers. Any product that may be evaluated in this article, or claim that may be made by its manufacturer, is not guaranteed or endorsed by the publisher.
